# Bis{2-[(2,4-dimethyl­phen­yl)imino­meth­yl]pyridine-κ^2^ *N*,*N*′}bis­(thio­cyanato-κ*N*)cadmium

**DOI:** 10.1107/S1600536812002772

**Published:** 2012-01-31

**Authors:** Mohammad Malekshahian, Mohamad Reza Talei Bavil Olyai, Behrouz Notash

**Affiliations:** a Department of Chemistry, Islamic Azad University, Karaj Branch, Karaj, Iran; b Department of Chemistry, Faculty of Science, Islamic Azad University, South Tehran Branch, Tehran, Iran; c Department of Chemistry, Shahid Beheshti University, G. C., Evin, Tehran 1983963113, Iran

## Abstract

The title compound, [Cd(NCS)_2_(C_14_H_14_N_2_)_2_], features crystallographic inversion symmetry with the Cd^II^ ion located on a centre of inversion. The Cd^II^ ion is six-coordinated in a slightly distorted octa­hedral geometry with the thiocyanate anions in axial positions. The angle between the benzene and pyridine rings is 69.64 (9)°. An inter­molecular C—H⋯S hydrogen bond stabilizes the crystal structure.

## Related literature

For the medicinal and pharmaceutical application of Schiff base compounds, see: Azza & Abu (2006 ▶); Dudek & Dudek (1966 ▶); Pandeya *et al.* (1999 ▶); Panneerselvam *et al.* (2005 ▶); Singh *et al.* (2006 ▶); Sridhar *et al.* (2001 ▶); Mladenova *et al.* (2002 ▶); Walsh *et al.* (1996 ▶). For the crystal structures of imino­pyridine complexes, see: Talei Bavil Olyai *et al.* (2008 ▶); Talei Bavil Olyai, Gholami Troujeni *et al.* (2010 ▶); Talei Bavil Olyai, Razzaghi Fard *et al.* (2010 ▶); Fallah Nejad *et al.* (2010 ▶); Loni *et al.* (2011 ▶).
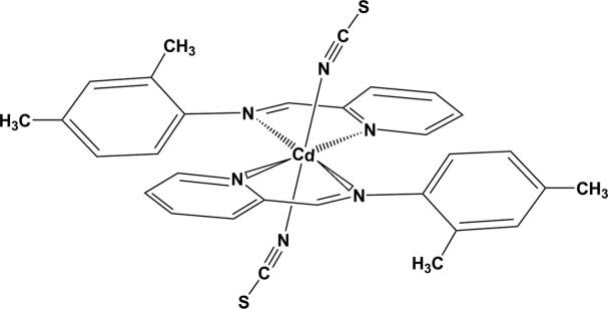


## Experimental

### 

#### Crystal data

[Cd(NCS)_2_(C_14_H_14_N_2_)_2_]
*M* *_r_* = 649.13
Orthorhombic, 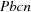

*a* = 11.285 (2) Å
*b* = 15.048 (3) Å
*c* = 17.576 (4) Å
*V* = 2984.7 (10) Å^3^
*Z* = 4
Mo *K*α radiation
μ = 0.90 mm^−1^
*T* = 298 K
0.45 × 0.4 × 0.4 mm

#### Data collection

Stoe IPDS II diffractometer
Absorption correction: numerical (*X-SHAPE* and *X-RED32*; Stoe & Cie, 2005 ▶)*T*_min_ = 0.406, *T*_max_ = 0.430
12952 measured reflections
4016 independent reflections
2589 reflections with *I* > 2σ(*I*)
*R*_int_ = 0.028

#### Refinement

*R*[*F* ^2^ > 2σ(*F* ^2^)] = 0.025
*wR*(*F* ^2^) = 0.075
*S* = 1.00
4016 reflections
181 parameters
H-atom parameters constrained
Δρ_max_ = 0.20 e Å^−3^
Δρ_min_ = −0.39 e Å^−3^

### 

Data collection: *X-AREA* (Stoe & Cie, 2005 ▶); cell refinement: *X-AREA*; data reduction: *X-AREA*; program(s) used to solve structure: *SHELXS97* (Sheldrick, 2008 ▶); program(s) used to refine structure: *SHELXL97* (Sheldrick, 2008 ▶); molecular graphics: *ORTEP-3 for Windows* (Farrugia, 1997 ▶); software used to prepare material for publication: *WinGX* (Farrugia, 1999 ▶).

## Supplementary Material

Crystal structure: contains datablock(s) I, global. DOI: 10.1107/S1600536812002772/bt5786sup1.cif

Structure factors: contains datablock(s) I. DOI: 10.1107/S1600536812002772/bt5786Isup2.hkl

Additional supplementary materials:  crystallographic information; 3D view; checkCIF report

## Figures and Tables

**Table 1:** Selected bond lengths (Å)

Cd1—N3	2.3032 (17)
Cd1—N1	2.3529 (14)
Cd1—N2	2.3708 (14)

Symmetry code: (i) 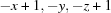.

**Table 2:** Hydrogen-bond geometry (Å, °)

*D*—H⋯*A*	*D*—H	H⋯*A*	*D*⋯*A*	*D*—H⋯*A*
C12—H12⋯S1^ii^	0.93	2.87	3.591 (2)	136

Symmetry code: (ii) 
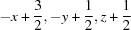
.

**Fig. 1.**
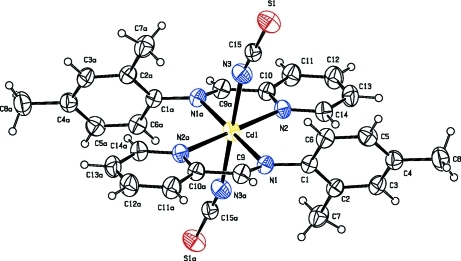
The molecular structure of the title molecule, with the atom-numbering scheme. Displacement ellipsoids are drawn at the 30% probability level [symmetry code:(a)-x + 1, -y, -z + 1].

**Fig. 2.**
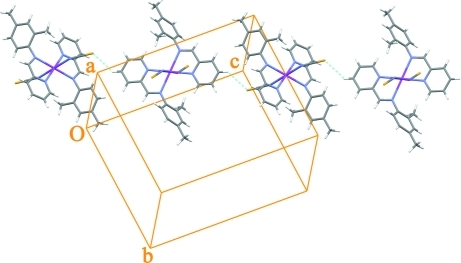
Packing diagram of the title compound showing intermolecular C—H···S hydrogen bonding.

## supplementary crystallographic information

### Comment

Nitrogen donor ligands particularly Schiff bases have been a subject of interest for chemists. Schiff bases form a class of compounds with azomethine group, which are usually synthesized from the condensation of primary amines and active carbonyl groups by elimination of water molecule. The Schiff bases and their metal complexes are important class of compounds in medicinal and pharmaceutical field (Azza & Abu, 2006; Dudek & Dudek, 1966; Pandeya *et al.*, 1999; Panneerselvam *et al.*, 2005; Singh *et al.*, 2006; Sridhar *et al.* 2001; Mladenova *et al.*, 2002; Walsh *et al.*, 1996).

Following our studies on the synthesis and structural determination of transition metal complexes with iminopyridine ligands by X-ray crystallography (Talei Bavil Olyai *et al.*, 2008; Talei Bavil Olyai, Gholami Troujeni *et al.*, 2010; Talei Bavil Olyai, Razzaghi Fard *et al.*, 2010; Fallah Nejad *et al.*, 2010; Loni *et al.*, 2011). We report herein the crystal structure of the title compound, a new cadmium(II) complex, (1), derived from the Schiff base ligand and thiocyanate. The title complex was synthesized by the reaction of Cd(CH_3_COO)_2_.2H_2_O with 2-[(2,4-dimethylphenyl)iminomethyl]- pyridine and KSCN in methanol as solution.

In the crystal structure of the title compound (Fig. 1), the cadmium(II) ion is six-coordinated in distorted octahedral geometry. Two Schiff base ligands coordinate the cadmium center as a bidentate ligand through the nitrogen atoms of imine group and pyridine ring. The Cd(II) ion is soft acidic metal center. According to symbiosis logic of Jorgensen, coordination of four electronegative nitrogen atoms of iminopyridine ligands have increased hardness of the cadmium ion and makes it a hard Lewis acid. Therefore, the Cd(II) ion prefers to bond to nitrogen atom of the ambidentate thiocyanate ligand.

The Cd—N_thiocyanate_ distances [2.3032 (17) Å] are notably shorter than the Cd—N_imine_ distances [2.3529 (1) Å] and Cd—N_pyridine_ [2.3708 (14) Å] (Table 1). The two imine linkages, C9—N1 [1.268 (2) Å], are both short, which is in the accepted range for carbon-nitrogen double bonds. Four donor nitrogen atoms of the iminopyridine ligands are absolutely planar with the Cadmium(II). In the title compound, coordination plane (containing the ligands backbone and the cadmium atom), and two thiocyanate ions are *trans* to each other. The angle between phenyl and pyridine rings are 69.64 (9) Å. In the crystal structure of the title compound an intermolecular C—H···S hydrogen bond (Table 2) stabilize crystal structure.

### Experimental

For the preparation of the title compound, a mixed solution of 2-[(2,4-dimethylphenyl)-iminomethyl]-pyridine (0.420 g, 2.00 mmol) and KSCN (0.195 g 2.00 mmol) in methanol (10 ml) was added slowly to a solution of Cd(CH_3_COO)_2_.2H_2_O (0.267 g, 1.00 mmol) in methanol (10 ml) and the resulting yellow solution was stirred for 45 min at room temperature, and then left to evaporate slowly at 3–5°C. After twenty days, yellow crystals of the title compound were isolated (yield; 0.426 g, 74.2%, m. p. 453 K).

### Refinement

All H atoms were positioned geometrically and refined as riding atoms with C—H=0.93(CH) and 0.96(CH_3_) Å and with *U*_iso_(H) = 1.2 (1.5 for methyl)*U*_eq_(C).

### Crystal data

**Table:**

[Cd(NCS)_2_(C_14_H_14_N_2_)_2_]	*F*(000) = 1320.0
*M**_r_* = 649.13	*D*_x_ = 1.445 Mg m^−^^3^
Orthorhombic, *P**b**c**n*	Mo *K*α radiation, λ = 0.71073 Å
Hall symbol: -P 2n 2ab	Cell parameters from 4016 reflections
*a* = 11.285 (2) Å	θ = 2.3–29.2°
*b* = 15.048 (3) Å	µ = 0.90 mm^−^^1^
*c* = 17.576 (4) Å	*T* = 298 K
*V* = 2984.7 (10) Å^3^	Block, yellow
*Z* = 4	0.45 × 0.4 × 0.4 mm

### Data collection

**Table:**

Stoe IPDS II diffractometer	4016 independent reflections
Radiation source: fine-focus sealed tube	2589 reflections with *I* > 2σ(*I*)
graphite	*R*_int_ = 0.028
Detector resolution: 0.15 mm pixels mm^-1^	θ_max_ = 29.2°, θ_min_ = 2.3°
rotation method scans	*h* = −15→13
Absorption correction: numerical shape of crystal determined optically	*k* = −20→18
*T*_min_ = 0.406, *T*_max_ = 0.430	*l* = −20→24
12952 measured reflections	

### Refinement

**Table:**

Refinement on *F*^2^	Secondary atom site location: difference Fourier map
Least-squares matrix: full	Hydrogen site location: inferred from neighbouring sites
*R*[*F*^2^ > 2σ(*F*^2^)] = 0.025	H-atom parameters constrained
*wR*(*F*^2^) = 0.075	*w* = 1/[σ^2^(*F*_o_^2^) + (0.0406*P*)^2^ + 0.0706*P*] where *P* = (*F*_o_^2^ + 2*F*_c_^2^)/3
*S* = 1.00	(Δ/σ)_max_ = 0.001
4016 reflections	Δρ_max_ = 0.20 e Å^−^^3^
181 parameters	Δρ_min_ = −0.39 e Å^−^^3^
0 restraints	Extinction correction: *SHELXL97* (Sheldrick, 2008), Fc^*^=kFc[1+0.001xFc^2^λ^3^/sin(2θ)]^-1/4^
Primary atom site location: structure-invariant direct methods	Extinction coefficient: 0.0048 (4)

### Special details

**Table:**

Geometry. All e.s.d.'s (except the e.s.d. in the dihedral angle between two l.s. planes) are estimated using the full covariance matrix. The cell e.s.d.'s are taken into account individually in the estimation of e.s.d.'s in distances, angles and torsion angles; correlations between e.s.d.'s in cell parameters are only used when they are defined by crystal symmetry. An approximate (isotropic) treatment of cell e.s.d.'s is used for estimating e.s.d.'s involving l.s. planes.
Refinement. Refinement of *F*^2^ against ALL reflections. The weighted *R*-factor *wR* and goodness of fit *S* are based on *F*^2^, conventional *R*-factors *R* are based on *F*, with *F* set to zero for negative *F*^2^. The threshold expression of *F*^2^ > σ(*F*^2^) is used only for calculating *R*-factors(gt) *etc*. and is not relevant to the choice of reflections for refinement. *R*-factors based on *F*^2^ are statistically about twice as large as those based on *F*, and *R*- factors based on ALL data will be even larger.

### Fractional atomic coordinates and isotropic or equivalent isotropic displacement parameters (Å^2^)

**Table:**

	*x*	*y*	*z*	*U*_iso_*/*U*_eq_	
Cd1	0.5000	0.0000	0.5000	0.05113 (8)	
S1	0.81414 (5)	0.10677 (4)	0.32032 (3)	0.08019 (19)	
N1	0.40527 (12)	0.08917 (9)	0.40838 (7)	0.0506 (3)	
N2	0.56541 (13)	0.09386 (10)	0.59987 (8)	0.0522 (3)	
N3	0.66504 (15)	0.03255 (13)	0.42813 (10)	0.0687 (4)	
C1	0.40926 (15)	0.18477 (11)	0.40648 (9)	0.0484 (4)	
C2	0.34254 (16)	0.23310 (12)	0.45844 (9)	0.0537 (4)	
C3	0.35215 (18)	0.32527 (12)	0.45528 (11)	0.0607 (5)	
H3	0.3075	0.3589	0.4892	0.073*	
C4	0.42425 (17)	0.36928 (12)	0.40456 (11)	0.0603 (5)	
C5	0.49090 (17)	0.31881 (14)	0.35509 (12)	0.0645 (5)	
H5	0.5413	0.3469	0.3208	0.077*	
C6	0.48400 (16)	0.22711 (14)	0.35565 (11)	0.0585 (5)	
H6	0.5295	0.1939	0.3219	0.070*	
C7	0.2607 (2)	0.18931 (16)	0.51445 (12)	0.0771 (6)	
H7A	0.2006	0.1569	0.4875	0.116*	
H7B	0.2241	0.2338	0.5457	0.116*	
H7C	0.3051	0.1492	0.5459	0.116*	
C8	0.4294 (2)	0.46981 (15)	0.40349 (16)	0.0888 (7)	
H8A	0.3775	0.4931	0.4419	0.133*	
H8B	0.4049	0.4911	0.3545	0.133*	
H8C	0.5090	0.4890	0.4134	0.133*	
C9	0.37889 (16)	0.04762 (12)	0.34795 (10)	0.0560 (4)	
H9	0.3556	0.0800	0.3054	0.067*	
C10	0.61622 (16)	0.04953 (12)	0.65736 (9)	0.0530 (4)	
C11	0.66386 (19)	0.09220 (14)	0.71998 (12)	0.0700 (5)	
H11	0.6992	0.0597	0.7588	0.084*	
C12	0.6583 (2)	0.18377 (15)	0.72401 (13)	0.0756 (6)	
H12	0.6895	0.2139	0.7656	0.091*	
C13	0.6062 (2)	0.22922 (14)	0.66575 (12)	0.0698 (5)	
H13	0.6016	0.2909	0.6670	0.084*	
C14	0.56032 (18)	0.18261 (13)	0.60500 (11)	0.0619 (5)	
H14	0.5243	0.2142	0.5658	0.074*	
C15	0.72622 (16)	0.06333 (12)	0.38293 (10)	0.0522 (4)	

### Atomic displacement parameters (Å^2^)

**Table:**

	*U*^11^	*U*^22^	*U*^33^	*U*^12^	*U*^13^	*U*^23^
Cd1	0.06666 (13)	0.05083 (12)	0.03589 (10)	−0.00527 (8)	−0.00409 (7)	0.00119 (7)
S1	0.0866 (4)	0.0912 (4)	0.0628 (3)	−0.0209 (3)	0.0033 (3)	0.0211 (3)
N1	0.0607 (8)	0.0512 (9)	0.0399 (7)	0.0054 (7)	0.0001 (6)	−0.0038 (6)
N2	0.0612 (9)	0.0500 (9)	0.0453 (8)	−0.0035 (7)	0.0000 (6)	−0.0037 (6)
N3	0.0716 (11)	0.0741 (11)	0.0603 (10)	−0.0121 (9)	0.0033 (8)	0.0023 (9)
C1	0.0585 (10)	0.0476 (10)	0.0390 (8)	0.0059 (7)	−0.0049 (7)	−0.0004 (7)
C2	0.0597 (10)	0.0555 (11)	0.0459 (9)	0.0052 (8)	0.0011 (8)	−0.0029 (8)
C3	0.0684 (11)	0.0553 (11)	0.0584 (10)	0.0112 (9)	−0.0002 (9)	−0.0086 (9)
C4	0.0667 (11)	0.0533 (11)	0.0609 (11)	−0.0013 (9)	−0.0121 (9)	0.0007 (9)
C5	0.0689 (12)	0.0657 (13)	0.0590 (11)	−0.0056 (10)	0.0020 (9)	0.0101 (10)
C6	0.0706 (12)	0.0607 (12)	0.0442 (9)	0.0069 (9)	0.0049 (8)	0.0000 (9)
C7	0.0892 (15)	0.0687 (13)	0.0733 (13)	0.0118 (12)	0.0293 (12)	0.0053 (11)
C8	0.1038 (19)	0.0544 (12)	0.108 (2)	−0.0100 (13)	−0.0139 (15)	0.0021 (13)
C9	0.0691 (11)	0.0571 (12)	0.0418 (9)	0.0060 (9)	−0.0044 (8)	−0.0015 (8)
C10	0.0618 (10)	0.0546 (12)	0.0426 (9)	0.0016 (8)	−0.0012 (8)	−0.0067 (8)
C11	0.0891 (14)	0.0677 (14)	0.0531 (10)	0.0060 (11)	−0.0140 (10)	−0.0143 (10)
C12	0.0940 (16)	0.0691 (15)	0.0636 (11)	−0.0024 (12)	−0.0115 (12)	−0.0225 (11)
C13	0.0852 (14)	0.0517 (11)	0.0727 (13)	−0.0019 (10)	0.0019 (11)	−0.0165 (10)
C14	0.0712 (13)	0.0538 (11)	0.0607 (11)	−0.0017 (9)	−0.0021 (10)	0.0008 (9)
C15	0.0588 (10)	0.0489 (9)	0.0491 (9)	−0.0032 (8)	−0.0094 (8)	0.0014 (8)

### Geometric parameters (Å, °)

**Table:**

Cd1—N3^i^	2.3032 (17)	C5—C6	1.382 (3)
Cd1—N3	2.3032 (17)	C5—H5	0.9300
Cd1—N1	2.3529 (14)	C6—H6	0.9300
Cd1—N1^i^	2.3529 (14)	C7—H7A	0.9600
Cd1—N2^i^	2.3708 (14)	C7—H7B	0.9600
Cd1—N2	2.3708 (14)	C7—H7C	0.9600
S1—C15	1.619 (2)	C8—H8A	0.9600
N1—C9	1.268 (2)	C8—H8B	0.9600
N1—C1	1.440 (2)	C8—H8C	0.9600
N2—C10	1.340 (2)	C9—C10^i^	1.466 (3)
N2—C14	1.340 (2)	C9—H9	0.9300
N3—C15	1.150 (2)	C10—C11	1.383 (2)
C1—C6	1.384 (3)	C10—C9^i^	1.466 (3)
C1—C2	1.389 (2)	C11—C12	1.381 (3)
C2—C3	1.392 (2)	C11—H11	0.9300
C2—C7	1.502 (3)	C12—C13	1.365 (3)
C3—C4	1.377 (3)	C12—H12	0.9300
C3—H3	0.9300	C13—C14	1.378 (3)
C4—C5	1.378 (3)	C13—H13	0.9300
C4—C8	1.514 (3)	C14—H14	0.9300
			
N3^i^—Cd1—N3	180.0	C6—C5—H5	119.5
N3^i^—Cd1—N1	97.43 (6)	C5—C6—C1	119.89 (18)
N3—Cd1—N1	82.57 (6)	C5—C6—H6	120.1
N3^i^—Cd1—N1^i^	82.57 (6)	C1—C6—H6	120.1
N3—Cd1—N1^i^	97.43 (6)	C2—C7—H7A	109.5
N1—Cd1—N1^i^	180.00 (5)	C2—C7—H7B	109.5
N3^i^—Cd1—N2^i^	91.58 (6)	H7A—C7—H7B	109.5
N3—Cd1—N2^i^	88.42 (6)	C2—C7—H7C	109.5
N1—Cd1—N2^i^	72.03 (5)	H7A—C7—H7C	109.5
N1^i^—Cd1—N2^i^	107.97 (5)	H7B—C7—H7C	109.5
N3^i^—Cd1—N2	88.42 (6)	C4—C8—H8A	109.5
N3—Cd1—N2	91.58 (6)	C4—C8—H8B	109.5
N1—Cd1—N2	107.97 (5)	H8A—C8—H8B	109.5
N1^i^—Cd1—N2	72.03 (5)	C4—C8—H8C	109.5
N2^i^—Cd1—N2	180.0	H8A—C8—H8C	109.5
C9—N1—C1	118.73 (15)	H8B—C8—H8C	109.5
C9—N1—Cd1	113.50 (12)	N1—C9—C10^i^	122.43 (16)
C1—N1—Cd1	124.85 (10)	N1—C9—H9	118.8
C10—N2—C14	117.64 (16)	C10^i^—C9—H9	118.8
C10—N2—Cd1	113.27 (11)	N2—C10—C11	122.37 (17)
C14—N2—Cd1	129.08 (12)	N2—C10—C9^i^	117.69 (15)
C15—N3—Cd1	161.83 (17)	C11—C10—C9^i^	119.94 (17)
C6—C1—C2	120.91 (16)	C12—C11—C10	119.1 (2)
C6—C1—N1	119.60 (16)	C12—C11—H11	120.4
C2—C1—N1	119.40 (15)	C10—C11—H11	120.4
C1—C2—C3	116.94 (17)	C13—C12—C11	118.8 (2)
C1—C2—C7	122.30 (17)	C13—C12—H12	120.6
C3—C2—C7	120.73 (17)	C11—C12—H12	120.6
C4—C3—C2	123.43 (18)	C12—C13—C14	119.2 (2)
C4—C3—H3	118.3	C12—C13—H13	120.4
C2—C3—H3	118.3	C14—C13—H13	120.4
C3—C4—C5	117.78 (18)	N2—C14—C13	122.92 (19)
C3—C4—C8	120.7 (2)	N2—C14—H14	118.5
C5—C4—C8	121.5 (2)	C13—C14—H14	118.5
C4—C5—C6	121.01 (19)	N3—C15—S1	179.04 (17)
C4—C5—H5	119.5		
			
N3^i^—Cd1—N1—C9	−97.15 (13)	N1—C1—C2—C3	178.39 (16)
N3—Cd1—N1—C9	82.85 (13)	C6—C1—C2—C7	−179.92 (19)
N2^i^—Cd1—N1—C9	−7.89 (12)	N1—C1—C2—C7	−3.3 (3)
N2—Cd1—N1—C9	172.11 (12)	C1—C2—C3—C4	−0.7 (3)
N3^i^—Cd1—N1—C1	102.54 (13)	C7—C2—C3—C4	−179.06 (19)
N3—Cd1—N1—C1	−77.46 (13)	C2—C3—C4—C5	−0.7 (3)
N2^i^—Cd1—N1—C1	−168.21 (13)	C2—C3—C4—C8	179.22 (19)
N2—Cd1—N1—C1	11.79 (13)	C3—C4—C5—C6	1.0 (3)
N3^i^—Cd1—N2—C10	79.80 (12)	C8—C4—C5—C6	−178.9 (2)
N3—Cd1—N2—C10	−100.20 (12)	C4—C5—C6—C1	0.0 (3)
N1—Cd1—N2—C10	177.10 (12)	C2—C1—C6—C5	−1.4 (3)
N1^i^—Cd1—N2—C10	−2.90 (12)	N1—C1—C6—C5	−178.07 (16)
N3^i^—Cd1—N2—C14	−101.74 (16)	C1—N1—C9—C10^i^	173.99 (15)
N3—Cd1—N2—C14	78.26 (16)	Cd1—N1—C9—C10^i^	12.4 (2)
N1—Cd1—N2—C14	−4.44 (17)	C14—N2—C10—C11	−0.9 (3)
N1^i^—Cd1—N2—C14	175.56 (17)	Cd1—N2—C10—C11	177.80 (15)
N1—Cd1—N3—C15	7.1 (5)	C14—N2—C10—C9^i^	179.59 (16)
N1^i^—Cd1—N3—C15	−172.9 (5)	Cd1—N2—C10—C9^i^	−1.76 (19)
N2^i^—Cd1—N3—C15	79.2 (5)	N2—C10—C11—C12	0.5 (3)
N2—Cd1—N3—C15	−100.8 (5)	C9^i^—C10—C11—C12	−179.9 (2)
C9—N1—C1—C6	−57.6 (2)	C10—C11—C12—C13	−0.2 (3)
Cd1—N1—C1—C6	101.74 (16)	C11—C12—C13—C14	0.3 (3)
C9—N1—C1—C2	125.68 (18)	C10—N2—C14—C13	0.9 (3)
Cd1—N1—C1—C2	−74.95 (18)	Cd1—N2—C14—C13	−177.48 (15)
C6—C1—C2—C3	1.7 (3)	C12—C13—C14—N2	−0.7 (3)

Symmetry codes: (i) −*x*+1, −*y*, −*z*+1.

### Hydrogen-bond geometry (Å, °)

**Table:**

*D*—H···*A*	*D*—H	H···*A*	*D*···*A*	*D*—H···*A*
C12—H12···S1^ii^	0.93	2.87	3.591 (2)	136.

Symmetry codes: (ii) −*x*+3/2, −*y*+1/2, *z*+1/2.
